# An application of weighted support vector machines to individualized second-line therapy selection after levodopa in Parkinson’s disease

**DOI:** 10.3389/fnhum.2026.1859515

**Published:** 2026-07-10

**Authors:** Cuong T. Pham, Ruth B. Schneider, Charles S. Venuto, Greta Smith, Zachary P. Brehm, Michael P. McDermott, Ashkan Ertefaie

**Affiliations:** 1 Department of Biostatistics and Computational Biology, University of Rochester, Rochester, NY, United States; 2 Center for Health and Technology, University of Rochester, Rochester, NY, United States; 3 Department of Neurology, University of Rochester, Rochester, NY, United States; 4 Department of Biostatistics, Epidemiology and Informatics, University of Pennsylvania, Philadelphia, PA, United States

**Keywords:** instrumental variable (IV) approach, machine learning, outcome weighted learning, Parkinson’s disease, personalized medicine, support vector machine

## Abstract

Parkinson’s disease (PD) is a progressive neurological disorder characterized by motor symptoms, with treatment responses varying widely across patients. To address the growing need for individualized treatment strategies, this paper introduces a practical framework for constructing optimal individualized treatment strategies using weighted support vector machines (SVMs). We construct an optimal rule as a function of patients’ clinical profiles and demographics using two methods: Outcome-Weighted Learning (OWL), which assumes no unmeasured confounders, and Instrumental Variable Outcome-Weighted Learning (IV-OWL), which accounts for potential unmeasured confounders. To illustrate the application of these methods, we use harmonized and curated clinical data from the NET-PD LS1 and PRECEPT/POSTCEPT studies to estimate the optimal second-line therapy decision rule for supplementing levodopa with either monoamine oxidase B (MAO-B) inhibitors or dopamine receptor agonists (DRA) based on patient characteristics. As an illustrative application, we analyzed two cohorts of patients who initiated second-line therapy within 90 and 180 days of baseline, with sample sizes of 84 and 121, respectively. Although the estimated improvements were not statistically significant, likely due in part to the small sample sizes, the learned IV-OWL rule showed improvement in annualized UPDRS III change compared with the observed treatment strategy and other static treatment strategies. This case study demonstrates the feasibility of applying weighted support vector machines to individualized second-line therapy selection in PD and illustrates the potential value of instrumental variable-based methods in addressing unmeasured confounders.

## Introduction

Parkinson’s disease (PD) is a progressive neurological disorder that affects over 1 million individuals in the United States (Marras et al., 2018). PD is characterized by motor symptoms, including tremor, rigidity, bradykinesia, gait disturbance, and postural instability, which are primarily related to the loss of dopamine-producing neurons in the substantia nigra. Current pharmacologic treatment strategies focus largely on replacing or enhancing dopaminergic activity to alleviate symptoms. Levodopa is the most effective therapy for improving motor function in PD (Murakami et al., 2023; Brooks, 2008). However, as the disease progresses, the effects of levodopa may wear off between doses, leading to increased motor and non-motor symptoms, and dyskinesias may develop (Fahn, 1989; Fahn et al., 2004). In the setting of wearing off, clinicians often supplement levodopa with an adjunctive medication, such as a monoamine oxidase B (MAO-B) inhibitor or a dopamine receptor agonist (DRA), among other options (Rabey et al., 2000; Zhang et al., 2023). Both classes are widely used in clinical practice, but they differ in mechanism of action, adverse effect profiles, and tolerability. Clear guidance on which class may be preferable for a given patient remains limited.

There is growing consensus that treatment strategies for Parkinson’s disease should be individualized to account for variability in treatment response and tolerability across patients (Titova and Chaudhuri, 2017). For example, in a study using micro-tablet formulations of levodopa/carbidopa, participants were able to personalize dosing more precisely, which was associated with self-reported improvements in the magnitude, frequency, and duration of dyskinesia (Grétarsdóttir et al., 2021). Similarly, personalization of non-pharmacologic interventions, such as exercise and deep brain stimulation, has been shown to improve motor symptoms and overall quality of life (Lützow et al., 2023; Swann et al., 2018). However, implementing personalized treatment strategies in routine PD care remains challenging. This creates a need for practical, data-driven methods that can support individualized decisions across a broader range of clinical settings.

In recent years, machine learning and deep learning approaches have been increasingly used to support the diagnosis and monitoring of PD progression. Methods such as random forests, support vector machines, and convolutional neural networks have been applied to diverse data sources, including imaging data, EEG signals, and speech recordings, for PD diagnosis and symptom assessment (Sharma et al., 2026). However, the application of machine learning and deep learning methods to individualized treatment strategies for PD remains limited. In a proof-of-concept study, Watts et al. showed that Asynchronous Advantage Actor-Critic (A3C), a deep reinforcement learning algorithm, could be used to personalize the timing and dosage of levodopa based on real-time motor fluctuation data collected from wearable sensors, with the goal of managing bradykinesia and dyskinesia (Watts et al., 2020). While deep reinforcement learning provides an automated framework for treatment personalization, its lack of interpretability makes it difficult to understand how individualized strategies are constructed. This limits its potential use in broader clinical practice, where transparency and clinical interpretability are important. Moreover, the study by Watts et al. was based on simulated data generated from the clinical literature rather than patient-level clinical trial data.

To address the limited interpretability of deep reinforcement learning methods and the need for practical guidance on individualized second-line therapy selection in PD, we use a practical framework for estimating individualized treatment strategies in PD based on weighted support vector machines (SVMs) (Cui and Tchetgen, 2021; Zhao et al., 2012). In this setting, outcome-weighted learning (OWL) is used to estimate an individualized treatment rule, which uses patient characteristics to recommend one of two treatment options with the goal of optimizing clinical outcomes. Compared with more complex machine learning approaches, such as A3C, OWL can provide a more interpretable decision rule. When implemented with a linear SVM, the resulting treatment rule can be expressed as a weighted combination of patient characteristics. This makes it easier for clinicians to understand how treatment recommendations are generated and to compare the learned rule with current clinical practice. Such interpretability may support more flexible and informed use of individualized treatment strategies in clinical settings.

Compared with other explainable optimal treatment regime methods, such as Q-learning or A-learning, OWL does not require direct specification of the full outcome regression model. This is useful when the relationship between patient characteristics, treatment, and clinical outcomes is difficult to model correctly. In addition, unlike prior proof-of-concept work that used simulated data, our study uses patient-level clinical trial data. This creates an important methodological challenge: treatment decisions in real data may be influenced by factors that are not fully captured in the available covariates. As a result, unmeasured confounding may be present and must be considered when estimating individualized treatment rules. As we show in this study, OWL can be extended to an instrumental variable framework, allowing individualized treatment rules to be estimated in settings where unmeasured confounding may be present.

Although OWL-based methods have been applied to estimate optimal individualized treatment regimes in various settings, to our knowledge, they have not previously been used to optimize the choice of second-line therapy for patients with PD. Therefore, we view the present study as a proof-of-concept demonstration that clinically interpretable, data-driven methods can be used to derive individualized treatment strategies for PD using existing patient-level clinical trial data. We consider two distinct methods for constructing optimal treatment strategies, depending on the underlying assumptions about the data. The first method assumes no unmeasured confounders, meaning there are no unobserved variables that affect both the treatment assignment mechanism and the outcome. This is an untestable assumption and often questionable, but it can vary depending on the study design, data quality, and domain knowledge. In contrast, the second method leverages instrumental variable (IV) techniques (Ertefaie et al., 2017; Baiocchi et al., 2014) that can provide unbiased causal effect estimates even in the presence of unmeasured confounders, provided that certain assumptions are met. An instrument is a pretreatment variable that is a predictor of treatment allocation, independent of unmeasured confounders and with no direct impact on the outcome (i.e., only affects the outcome through affecting the treatment allocation). We also introduce an estimator to evaluate the effectiveness of a given treatment strategy.

To demonstrate these methods, we applied them to harmonized and curated clinical data from the NET-PD LS1 and PRECEPT/PostCEPT clinical trials (Parkinson Study Group PRECEPT Investigators, 2007; Kieburtz et al., 2015). Our goal was to estimate an individualized treatment decision rule to optimize motor function by guiding the choice of whether to supplement levodopa with an MAO-B inhibitor or a DRA based on the patient’s characteristics. While both second-line strategies have shown benefits, responses may vary based on a patient’s characteristics, highlighting the need to construct an optimal individualized treatment decision rule for this scenario. This paper aims to introduce OWL and Instrumental Variable OWL (IV-OWL) as practical methods for learning individualized treatment strategies in PD, using the choice of adjunctive therapy after levodopa initiation as a clinically relevant case study.

## Methods

### Data source

We utilize data from two randomized clinical trials involving participants with early PD to learn the individualized treatment strategies regarding the choice of second-line therapy following initial treatment with levodopa.

NET-PD LS1 (NCT00449865) was a multicenter randomized, double-blind, placebo-controlled clinical trial designed to evaluate the efficacy of creatine in slowing long-term clinical decline in patients with PD (Kieburtz et al., 2015). From March 2007 to May 2010, the trial enrolled 1,741 participants across 45 sites in the United States and Canada, with follow-up continuing through September 2013. The participants had early-stage PD, diagnosed within the previous 5 years, and had been receiving levodopa, a DRA, or both for at least 90 days but no longer than 2 years. Other prescribed PD therapies were permitted to continue during the study. Participants were scheduled to be followed for a minimum of 5 years, with some followed for up to 8 years, depending on their enrollment date. The trial was terminated early for futility following a planned interim analysis after 55% of participants were eligible for a 5-year follow-up visit.

During the first year, follow-up visits occurred every 3 months and transitioned to every 6 months thereafter. Demographics were collected at baseline, and medications, weight, blood pressure, and adverse events were collected at all follow-up visits. The Unified Parkinson’s Disease Rating Scale (UPDRS) (Fahn, 1987) was collected at baseline, Month 3, Year 1, and annually thereafter. Participants were allowed to adjust or change their concomitant treatments during the study, and these modifications were also recorded.

The Parkinson’s Research Examination of CEP-1347 Trial (PRECEPT; NCT00040404) was another multicenter randomized, double-blind, placebo-controlled clinical trial designed to evaluate the effects of a mixed-lineage kinase inhibitor in patients with early PD. This trial enrolled 806 participants at 65 sites in the US and Canada. At baseline, participants had to have untreated PD with a modified Hoehn and Yahr stage < 2.5 and not be expected to require dopaminergic treatment for at least 3 months. On May 12, 2005, after an average of 21.4 months of follow-up, the study was terminated following a planned interim analysis that showed that it was futile to continue due to a lack of efficacy. In February 2006, PRECEPT participants were invited to join a prospective observational follow-up study called PostCEPT. During PRECEPT, participants were followed every 3 months, with data collected on demographics, medications, vital signs, adverse events, and the UPDRS Parts I-IV. In PostCEPT, follow-up continued annually. In both PRECEPT and PostCEPT, dopaminergic treatment could be added when participants showed disability that required dopaminergic treatment. Participants could change the type of dopaminergic treatment or the dosage at any time during follow-up. Non-dopaminergic PD treatments were also permitted and tracked throughout the study. De-identified data were used in the analysis, which we accessed on May 15, 2024.

### Modeling study design

Our target population included PD patients whose first-line therapy was levodopa, and the second-line therapy was either a DRA or an MAO-B inhibitor. In the NET-PD LS1 dataset, we focused on participants initially treated with levodopa alone who added either a DRA or an MAO-B inhibitor during the study. Participants who entered the trial treated with a DRA or an MAO-B inhibitor were excluded. From PRECEPT/PostCEPT, we included participants for whom levodopa was their first dopaminergic therapy and for whom treatment with a DRA or an MAO-B inhibitor was subsequently initiated during the PostCEPT follow-up period. In all studies, the choice of second-line therapy was determined by the clinician at each site, making comparisons between the second-line therapies potentially susceptible to confounding.

To construct the optimal treatment strategies, we employed two methods. The first method, Outcome-Weighted Learning (OWL), assumes that no unmeasured confounders influence the timing of the second-line therapy initiation. The second method, Instrumental Variable Outcome-Weighted Learning (IV-OWL), accounts for potential unmeasured confounders, which may impact when patients start second-line therapy. These two approaches allowed us to explore the same clinical question, whether a given patient profile is more likely to benefit from DRA or MAO-B inhibitor therapy, but under different assumptions about unmeasured confounders.

### Definition of baseline

In both the NET-PD LS1 and PRECEPT studies, participants often began second-line therapy between two in-person visits, when clinical data were not recorded. For the purposes of this analysis, we defined the visit immediately preceding initiation of second-line therapy as the analytic “baseline” because it provides the closest available pre-initiation clinical snapshot for informing the treatment decision. Since “baseline” information should closely relate to the treatment decision, we imposed time restrictions of 90 and 180 days to limit the gap between the analytic “baseline” visit and second-line therapy initiation.

### Outcome

The outcome of interest was the annualized change in the UPDRS III motor score approximately 1 year after the initiation of second-line therapy. The UPDRS III (Movement Disorder Society Task Force on Rating Scales for Parkinson’s Disease, 2003) consists of 14 clinician-scored items that measure a patient’s motor function. The score ranges from 0 to 108, with higher scores representing greater symptom severity.

The outcome was measured approximately a year after the initiation of the second-line therapy. We used the outcomes at the visit immediately after “baseline,” provided the time between the start of second-line therapy and the visit was at least 120 days. If this condition was not met, the outcomes at the second visit after “baseline” were used. The annualized change in the UPDRS III score was calculated as the difference between the UPDRS III scores after and before second-line therapy initiation, divided by the time (in years) between these two measurements.

### Covariates included in the optimal decision rule

The decision rule includes the following covariates: sex, body mass index (BMI), UPDRS I, II, and III scores, presence of dyskinesias (yes/no) and presence of motor fluctuations (yes/no) from UPDRS IV, and the presence of nausea (yes/no) and insomnia (yes/no) from adverse event data, as assessed at “baseline”; and age, years since PD diagnosis, years of levodopa exposure, and levodopa dosage at the actual time of initiation of second-line therapy.

### Instrumental variable

**Algorithm 1:**
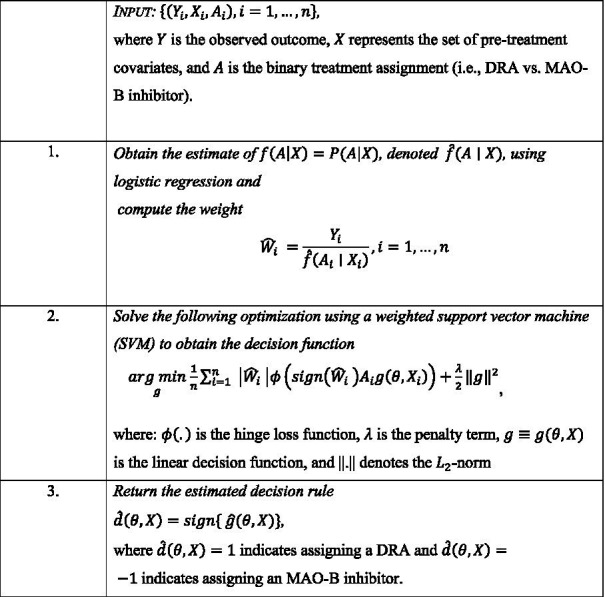
Pseudo algorithm for the proposed weighted learning under the assumption of no unmeasured confounders

**Figure 1:**
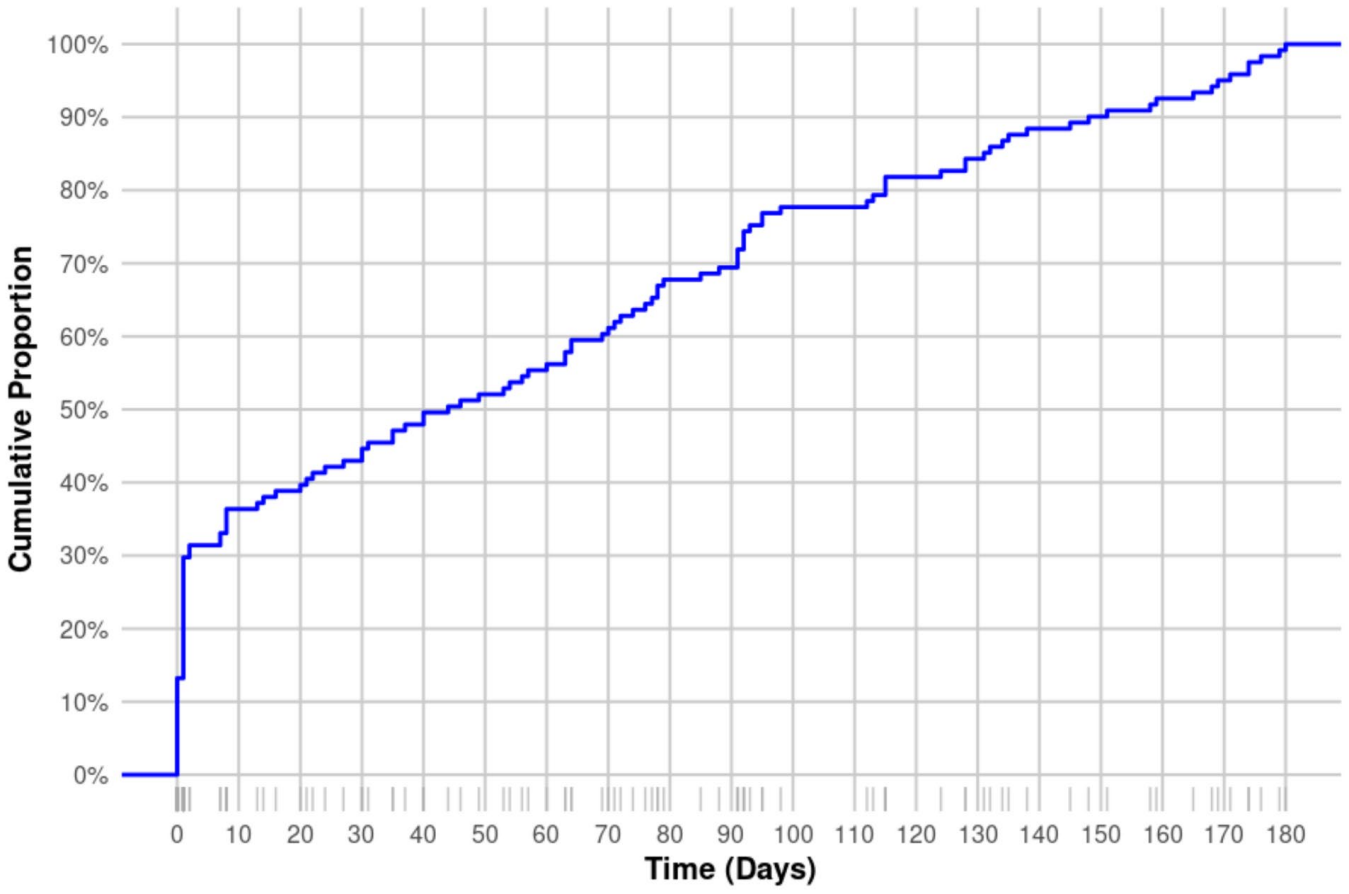
Cumulative proportion of participants as a function of the number of days between “baseline” and the start of their second-line therapy.

Since, for some participants, clinical variables (e.g., adverse events and UPDRS) are not recorded close to the time of second-line therapy initiation (90 or 180 days between events), it is plausible that the assumption of no unmeasured confounders is violated. Figure 1 presents the cumulative proportion of participants starting second-line therapy based on the number of days elapsed from “baseline.” Notably, only 12% of participants initiated second-line therapy on the same day as their “baseline” visit at which clinical variables were assessed, while 60% started at least 20 days after “baseline.” A common approach to address the potential unmeasured confounders problem is to use an instrumental variable (IV) (Ertefaie et al., 2017; Baiocchi et al., 2014). A valid IV is a pre-treatment variable that satisfies the following three conditions: (A1) it is associated with the treatment; (A2) it only affects the outcome through the treatment (exclusion restriction assumption); and (A3) it is not associated with unmeasured confounders after conditioning on measured confounders. Thus, in practical terms, a valid IV should influence which treatment is prescribed but should not have a direct effect on the outcome. An important first step in instrumental variable (IV) analysis is identifying a valid IV, which can be challenging because, unlike A1, both A2 and A3 cannot be tested using the observed data. Provider preference, which reflects naturally occurring variation in clinical practice, has been widely used as an IV in clinical settings (Ertefaie et al., 2017; Baiocchi et al., 2014). Specifically, the IV can be defined as the treatment (e.g., DRA vs. MAO-B inhibitor) that has a higher chance of being prescribed by a particular provider or center. Following a similar idea, in our study, we use center-level (i.e., center ID) treatment preference as the basis for a binary IV. We fit a mixed-effects logistic regression model with center ID as a random effect, the “baseline” covariates included in the decision rule as fixed effects, and treatment assignment (DRA vs. MAO-B inhibitor) as the outcome variable. The median of the predicted intercepts was used as the cutoff to create the binary IV, which represents treatment assignment tendency (or preference) in each center (Ertefaie et al., 2018).

### Statistical methods

#### Under the assumption of no unmeasured confounders

The optimal decision rule was constructed using a weighted supporting vector machine (SVM) method (Zhao et al., 2012). Conceptually, this method estimates a treatment rule that assigns DRA or MAO-B inhibitor therapy based on a linear combination of patient characteristics measured at the analytic “baseline” with the goal of optimizing the subsequent motor outcome. In addition to the assumption of no unmeasured confounding, we assume two standard causal assumptions: consistency and positivity. Consistency requires that the observed outcome under the treatment received corresponds to the potential outcome under that treatment. Positivity requires that, within levels of the observed covariates, each treatment option has a positive probability of being assigned. Let D denote a class of parametric decision rules defined as a function of a linear combination of “baseline” covariates. Specifically, let $g\equiv g(\theta ,x)=\theta^{\prime }x$ be the linear decision function, where θ is a vector of unknown parameters. Then D={d(θ,x):d(θ,x)=sign{g(θ,x)} , where sign{g(θ,x)}=1 if g(θ,x)≥0 and sign{g(θ,x)}=−1 otherwise. Let X be the vector of observed pre-treatment covariates, Y the outcome of interest to optimize, and A∈{−1,1} the binary treatment assigned, where −1 indicates assignment to an MAO-B inhibitor and 1 indicates assignment to a DRA. Under the standard causal assumptions, we can define an optimal treatment strategy as:


$$\underset{D}{\arg\;\min\;}E[\frac{\mathrm{AYI}\{A\neq D(\theta ,X)\}}{f(A|X)}]$$ (1)

The nuisance function f(A∣X) is estimated using a logistic regression model that includes treatment assignment (DRA vs. MAO-B inhibitor) as the dependent variable and the covariates X as the independent variables. After plugging in the corresponding estimated nuisance parameter $\hat{f}(A\;|\;X)$ in Equation 1, we use the method of Zhao et al. (2012), which utilizes the machine learning method weighted SVM to solve the resulting optimization problem. The process of finding d(θ,X) is detailed in Algorithm 1. In the second step of the algorithm, ∣∣g∣∣ denotes the $L_{2}$ -norm of g and λ is the penalty term obtained using cross-validation. The term $\frac{\lambda }{2}{\left\|g\right\|}^{2}$ penalizes the complexity of the decision function g(θ,x) to avoid overfitting. In our paper, Algorithm 1 was implemented in R version 4.3.1. The optimization was performed using the WeightSVM package, version 1.7–16 (Xu, 2024). The computation was performed on a 13th Gen Intel(R) Core i9-13900H (2.60 GHz) processor and 32GB memory. The full Monte Carlo cross-validation procedure with 360 repetitions took approximately 22.78 s.

### In the presence of unmeasured confounders

In the presence of unmeasured confounders, an instrumental variable can be used to develop an optimal individualized strategy. Let Z∈{−1,1} be the binary instrumental variable (IV). Denote δ(X)=P(A=1∣Z=1,X)−P(A=1∣Z=−1,X) and f(Z∣X)=P(Z∣X) . Under the assumption A1 – A3, along with consistency and positivity, we can define an optimal strategy as


$$\underset{D}{\arg\;\min\;}E[\frac{\text{AZYI}\{A\neq D(\theta ,X)\}}{\delta (X)f(Z|X)}]$$ (2)

Similar to the case where unmeasured confounders do not exist, we can minimize Equation 2 using weighted SVM (Cui and Tchetgen, 2021). The process of finding d(θ,X) is detailed in Algorithm 2. The nuisance functions δ(X) and f(Z∣X) in Equation 2 are estimated using logistic regression models. To estimate δ(X) , we fit a logistic regression model that includes treatment assignment (DRA vs. MAO-B inhibitor) as the dependent variable, and the instrumental variable Z as well as the covariates X as the independent variables. We estimate P(A=1∣Z=1,X) by setting Z=1 and P(A=1∣Z=−1,X) by setting Z=−1 . The estimate of δ(X) is the difference between the two estimated probabilities. Similarly, f(Z∣X) is fitted using the instrumental variable Z as the dependent variable and covariates X as independent variables. Like Algorithm 1, Algorithm 2 was also implemented in R version 4.3.1 and the optimization was performed using the WeightSVM package, version 1.7–16 (Xu, 2024). The computation was performed on a 13th Gen Intel(R) Core i9-13900H (2.60 GHz) processor and 32GB memory. The full Monte Carlo cross-validation procedure with 360 repetitions took approximately 30.74 s.

### Estimation of the value functions

An important step in constructing an optimal strategy is to quantify its quality. We define a value function of any given decision rule as the mean counterfactual outcome under that rule. In this paper, we propose to use the doubly robust estimator ${\overset{ˆ}{V}}_{\mathrm{MR}}(D)$ , whose form is given in Section A of the Appendix. This estimator also utilizes instrumental variables to produce an unbiased estimator of the value function in the presence of unmeasured confounders. An important property of this estimator is the doubly robust property (Wang and Tchetgen Tchetgen, 2018). It will be a consistent estimator if either the propensity score or the outcome regression model is correctly specified. Also, when both functions are correctly specified, the resulting value function will be the most efficient estimator. We use semiparametric efficiency theory to obtain Wald-type confidence intervals for the value function given by:


$${\overset{ˆ}{V}}_{\mathrm{MR}}(D)\pm 1.96\sqrt{\overset{ˆ}{V}\{V_{\mathrm{MR}}(D)\}},$$

where V denotes the variance operator.

**ALGORITHM 2:**
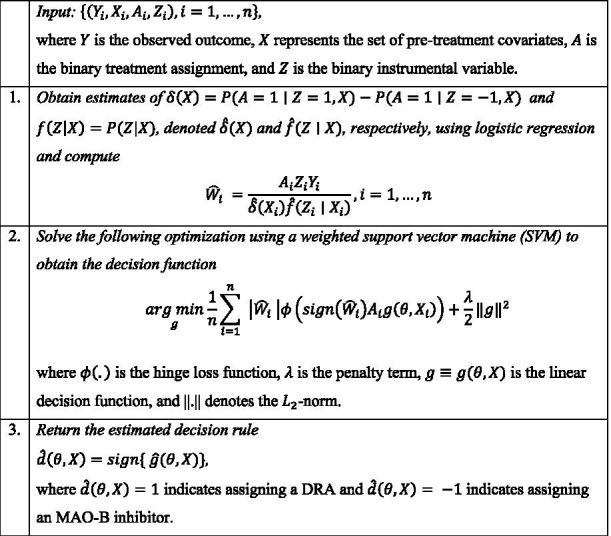
Pseudo algorithm for the proposed weighted learning in the presence of unmeasured confounders.

We compared the constructed optimal strategy with decision rules that assign all participants to DRA (DRA-only) or all participants to an MAO-B inhibitor (MAOB-only). We also considered the value function for the decision rule that yielded the observed outcomes in the data. This is an important quantity as it reflects the value of the treatment strategies followed by the clinicians that participated in the NET-PD LS1 and PRECEPT trials – we refer to this as the data-generating strategy. We report the difference between estimated value functions under the optimal, DRA-only, and MAOB-only strategies and the data-generating strategy. A large positive difference indicates that the considered strategy (i.e., optimal strategy, DRA-only, or MAOB-only) is better than the data-generating strategy. To avoid overfitting, we employed a Monte Carlo cross-validation process (Bates et al., 2024; Stone, 2018). We randomly split the dataset into a training set ( 70% of the data) and a test set ( 30% of the data). We used the training set to train our weighted SVM model and estimated the value function based on the test set. We repeated the whole process 360 times to obtain approximate distributions of the value function and the coefficients of the linear decision rules.

## Results

### Demographics

We defined “baseline” covariates in two ways: those measured within 90 or 180 days prior to the initiation of second-line therapy. Table 1 summarizes the “baseline” characteristics of the 84 participants who initiated second-line therapy within 90 days of “baseline,” the additional 37 participants who started this therapy within 91–180 days, and the total sample of 121 participants who started this therapy within 180 days of “baseline.” Among the participants who began second-line therapy within 90 days of “baseline,” the average age was 64.4 years (SD = 8.8), and the mean time since their PD diagnosis was 2.1 years (SD = 1.7). Most participants were male (71.4%) and identified as White (92.9%). On average, they had been taking levodopa for 1.7 years (SD = 1.2) before starting supplemental treatment, with 53.6% selecting DRA as the additional drug. In comparison, the added participants who started therapy within 91–180 days of “baseline” had a slightly lower average daily levodopa dosage (443.2 mg/day vs. 486.0 mg/day) and a higher prevalence of motor fluctuations (43.2% vs. 31.0%).

**Table 1:** “Baseline” characteristics of participants who initiated second-line therapy within 90 days, of participants added when the cutoff was extended to 180 days, and the combined sample of participants who initiated second-line therapy within 180 days.

Characteristics	Within 90 Days (*n =* 84)	91–180 Days (*n =* 37)	Within 180 Days (*n =* 121)
Male sex	60 (71.4%)	29 (78.4%)	89 (73.6%)
White race	78 (92.9%)	34 (91.9%)	112 (92.6%)
Body Mass Index (kg/m^2^)	27.7 (4.9)	26.8 (3.6)	27.4 (4.6)
Age (years)	64.4 (8.8)	65.6 (9.2)	64.8 (8.9)
Time since PD diagnosis (years)	2.1 (1.7)	2.2 (1.7)	2.2 (1.6)
Levodopa dosage (mg/day)	486.0 (290.8)	443.2 (230.7)	472.9 (273.6)
Duration of levodopa use (years)	1.7 (1.2)	2.1 (1.2)	1.8 (1.2)
Dopamine receptor agonist as second-line therapy	45 (53.6%)	16 (43.2%)	61 (50.4%)
Unified Parkinson’s Disease Rating Scale (UPDRS) Scores			
UPDRS I	1.3 (1.3)	1.3 (1.3)	1.3 (1.3)
UPDRS II	7.4 (4.1)	8.0 (5.5)	7.6 (4.5)
UPDRS III	18.4 (8.9)	20.6 (10.2)	19.1 (9.3)
Dyskinesias	10 (11.9%)	4 (10.8%)	14 (11.6%)
Motor fluctuations	26 (31.0%)	16 (43.2%)	42 (34.7%)
Nausea	12 (14.3%)	7 (18.9%)	19 (15.7%)
Insomnia	36 (42.9%)	13 (35.1%)	49 (40.5%)

Binary characteristics are presented as *n* (%), and continuous characteristics are presented as mean (standard deviation).

Characteristics recorded at the time of treatment initiation are presented in Table 2. These values do not differ substantially from those reported at the “baseline” visit.

**Table 2:** Characteristics at the initiation of second-line therapy of participants who initiated second-line therapy within 90 days, of participants added when the cutoff was extended to 180 days, and the combined sample of participants who initiated second-line therapy within 180 days.

Characteristics	Timing of initiation of second-line therapy		
	Within 90 days (*n =* 84)	91–180 days (*n =* 37)	Within 180 days (*n =* 121)
Age (years)	64.5 (8.8)	65.9 (9.2)	64.9 (8.9)
Time since PD diagnosis (years)	2.2 (1.7)	2.6 (1.7)	2.3 (1.7)
Levodopa dosage (mg/day)	486.6 (308.8)	462.2 (298.7)	479.1 (304.7)

Values are presented as mean (SD).

### Optimal treatment strategy

Table 3 shows the improvement in the mean annualized change in the UPDRS III score for various treatment strategies relative to the data-generating strategy. The IV-OWL strategy provided the greatest improvement. For participants who initiated second-line therapy within 90 days of “baseline,” the IV-OWL strategy was estimated to improve the mean UPDRS III score by 1.18 points more than the data-generating strategy over 1 year. When considering participants who started second-line therapy within 180 days of “baseline,” the relative improvement remained at a mean 1.10 points per year. However, while the mean annualized changes in UPDRS III for the IV-OWL strategies were positive, their confidence intervals included 0, indicating that the improvement was not statistically significant. In contrast, the OWL strategy was estimated to result in a mean improvement in UPDRS III score that was approximately 3 points lower than that of the data-generating strategy, indicating worse outcomes. Among specific treatment strategies, the MAOB-only approach outperformed the DRA-only approach but still fell short compared to the IV-OWL strategy. Similarly to the IV-OWL strategy, the confidence intervals for the mean annualized change in UPDRS III under the OWL strategy included 0, indicating that there is not enough evidence to conclude that the OWL strategy is significantly worse than the data-generating strategy.

**Table 3:** Estimated improvement of the value function for different treatment strategies relative to the data-generating strategy.

Threshold^1^	OWL optimal^2^	IV OWL optimal^3^	MAOB only^4^	DRA-only^5^	Sample size
90	−3.11 (−22.23, 16.01)	1.18 (−13.02, 15.38)	0.60 (−10.48, 11.68)	−1.67 (−16.83, 13.49)	84
180	−3.00 (−16.44, 10.44)	1.10 (−7.82, 10.02)	−0.02 (−7.42, 7.38)	−1.93 (−14.71, 10.85)	121

Values are presented as Mean (95% CI). ^1^The number of days from “baseline” within which patients initiated second-line therapy. A threshold value of 90 means the sample includes only patients who started second-line therapy within 90 days, while a threshold value of 180 includes only those who initiated therapy within 180 days. ^2^The optimal treatment strategy constructed using the OWL method. ^3^The optimal treatment strategy constructed using the IV-OWL method. ^4^Strategy where all patients receive monoamine oxidase B (MAO-B) inhibitors as second-line treatment. ^5^Strategy where all patients receive dopamine receptor agonists (DRAs) as second-line treatment.

Table 4 presents the estimated coefficients for the linear decision functions that define the OWL and IV-OWL strategies. The estimated coefficients provide insight into how the optimal strategies make treatment recommendations. The sign of each coefficient indicates its influence on treatment assignment: a positive coefficient suggests that as the covariate increases, the optimal strategy is more likely to recommend adding an MAO-B inhibitor to levodopa. For example, the coefficient for dyskinesias in the IV-OWL linear decision rule is 0.20, indicating that participants with dyskinesias are more likely to be prescribed an MAO-B inhibitor. In contrast, the coefficient for BMI is −0.22, suggesting that participants with a higher BMI are more likely to receive a DRA. This interpretability is a key advantage of the OWL and IV-OWL methods over black-box machine learning approaches, such as reinforcement learning.

**Table 4:** Coefficients of the linear decision rules for the IV-OWL and OWL strategies.

Covariates	IV-OWL optimal strategy		OWL optimal strategy	
	90 days	180 days	90 days	180 days
Intercept	0.08 (0.44)	0.05 (0.31)	0.05 (0.45)	−0.11 (0.6)
UPDRS I score	0.12 (0.81)	−0.05 (0.4)	−0.05 (0.63)	−0.15 (0.53)
UPDRS II score	−0.06 (0.72)	−0.09 (0.51)	−0.08 (0.65)	−0.03 (0.61)
UPDRS III score	−0.15 (0.81)	−0.02 (0.43)	−0.04 (0.68)	0.12 (0.73)
Years since PD diagnosis	0.03 (0.73)	0.03 (0.49)	−0.25 (0.94)	−0.13 (0.61)
Daily levodopa dosage	−0.07 (0.7)	−0.19 (0.64)	0.33 (0.67)	0.02 (0.7)
Male sex	0.38 (1.02)	0.40 (0.95)	−0.27 (0.93)	−0.10 (0.54)
Age	0.01 (0.73)	−0.03 (0.34)	0.03 (0.74)	0.01 (0.47)
Body mass index	−0.27 (0.68)	−0.22 (0.6)	0.17 (0.85)	0.08 (0.48)
Dyskinesias	0.19 (0.93)	0.20 (0.71)	−0.02 (0.94)	0.16 (0.91)
Motor fluctuations	−0.03 (0.78)	−0.03 (0.44)	−0.03 (0.73)	0.00 (0.51)
Nausea	−0.12 (0.74)	−0.28 (0.74)	−0.02 (0.65)	−0.31 (0.91)
Insomnia	0.10 (0.74)	0.32 (0.69)	0.03 (0.66)	0.21 (0.5)
Years of levodopa use	0.39 (1.12)	0.26 (0.74)	0.39 (0.99)	0.11 (0.65)

Values are presented as Estimate (SE). A negative coefficient indicates a preference for the dopamine receptor agonist (DRA), while a positive coefficient indicates a preference for the MAO-B inhibitor.

Table 4 also reports the standard error for each coefficient, which indicates whether there is sufficient evidence that a covariate influences the decision rule. Standard errors allow for the construction of confidence intervals. Specifically, a 95% confidence interval for a coefficient is calculated as:


Estimated Coefficient±1.96×Standard Error

If a confidence interval for a coefficient does not include 0, we can conclude that the corresponding covariate plays a role in the treatment assignment. However, in our case, the standard errors for all coefficients are relatively large, which results in wide confidence intervals. For example, although the coefficient for dyskinesia is 0.20, its standard error is 0.71, and the corresponding 95% confidence interval is between −1.19 and 1.59, which contains 0. Therefore, we cannot conclude that dyskinesia is an important variable in the true optimal decision rule, or that the true optimal rule would assign an MAO-B inhibitor to patients with dyskinesia. Similarly, none of the estimated coefficients are statistically different from zero. We are unable to conclude that any covariate we considered in our models plays a key role in defining the optimal treatment strategy.

**Figure 2:**
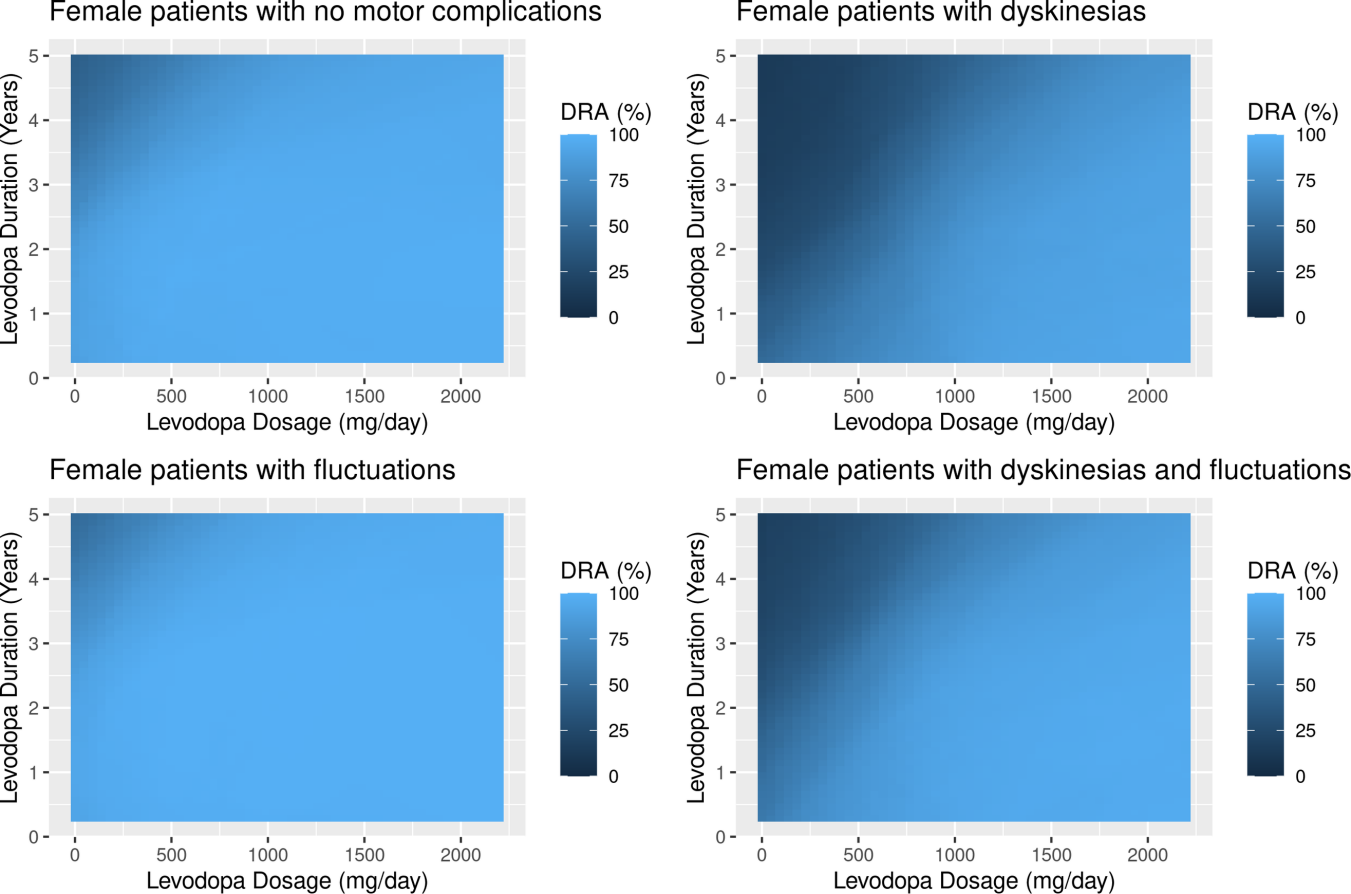
Heat maps showing the percentage of DRA treatment assignment for female subgroups based on levodopa dosage (mg/day), levodopa duration (years), and motor complications. Other covariates were held fixed at their reference or mean values. The decision rule is estimated using the IV-OWL method, with lighter blue indicating a higher percentage of DRA assignment.

**Figure 3:**
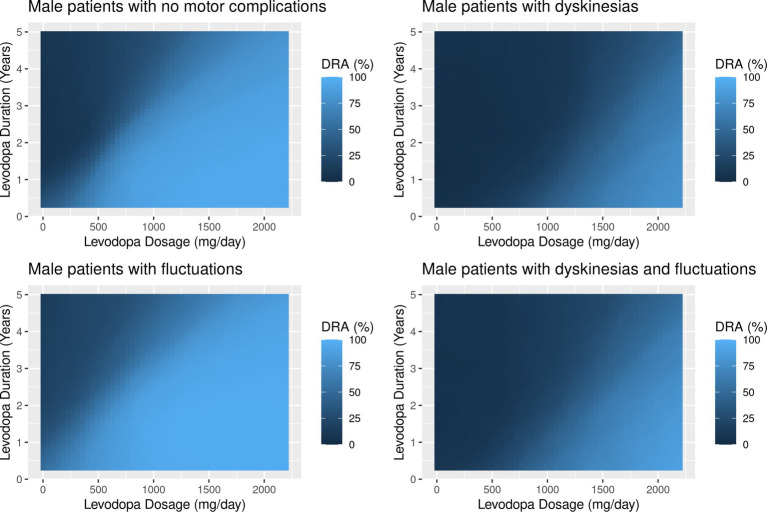
Heat maps showing the percentage of DRA treatment assignment for male subgroups based on levodopa dosage (mg/day), levodopa duration (years), and motor complications. Other covariates were held fixed at their reference or mean values. The decision rule is derived using the IV-OWL method, with lighter blue indicating a higher percentage of DRA assignment.

**Figure 4:**
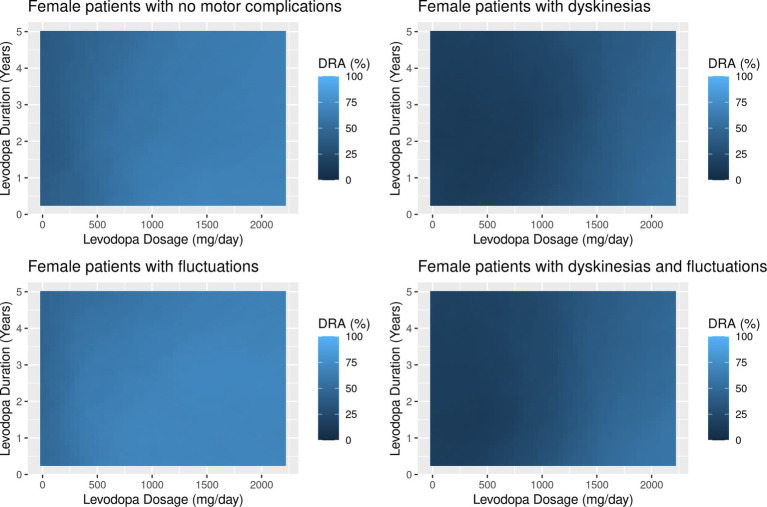
Heat maps showing the percentage of DRA treatment assignment for female subgroups based on levodopa dosage (mg/day), levodopa duration (years), and motor complications. Other covariates were held fixed at their reference or mean values. The decision rule is estimated using the OWL method, with lighter blue indicating a higher percentage of DRA assignment.

**Figure 5:**
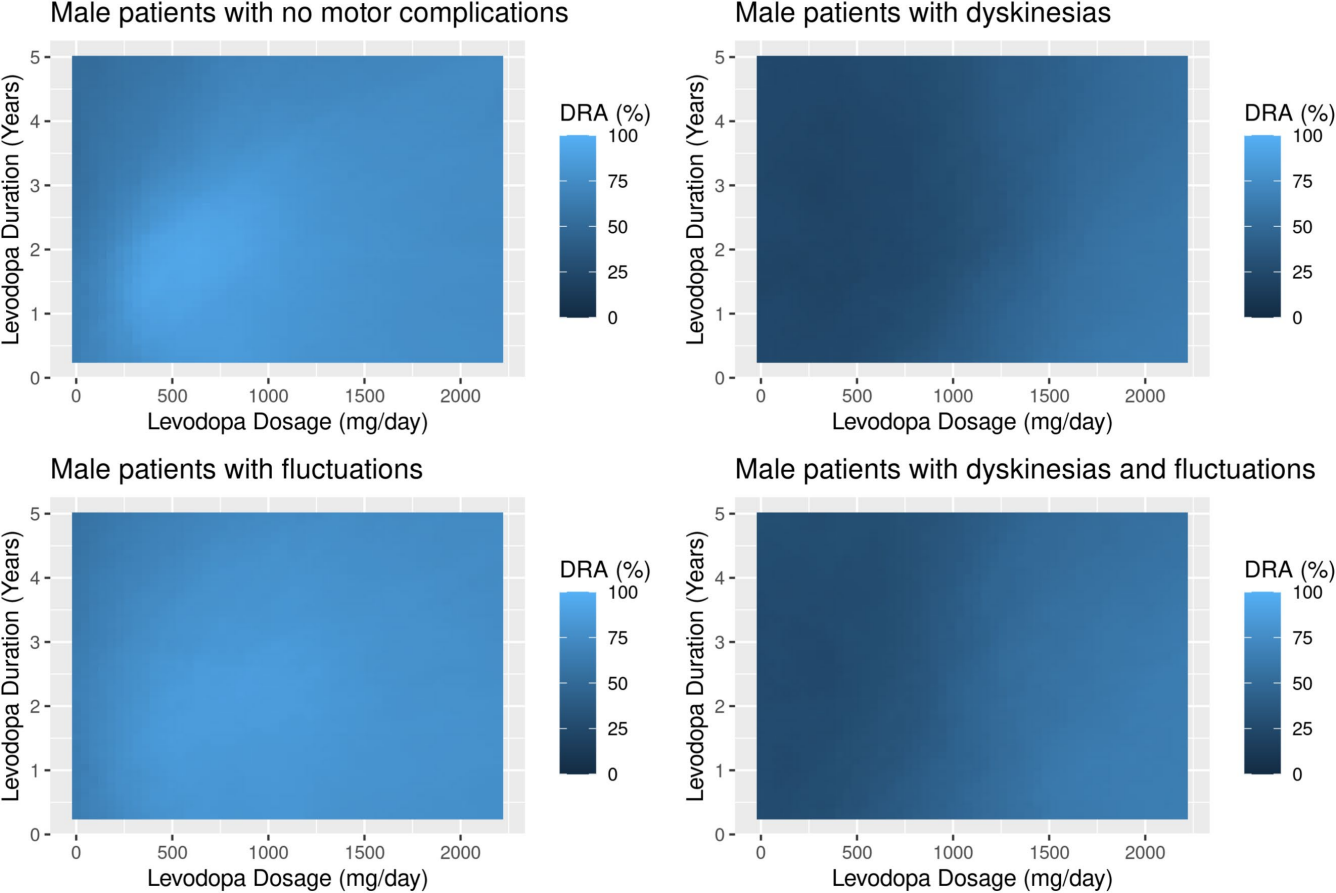
Heat maps showing the percentage of DRA treatment assignment for male subgroups based on levodopa dosage (mg/day), levodopa duration (years), and motor complications. Other covariates were held fixed at their reference or mean values. The decision rule is estimated using the OWL method, with lighter blue indicating a higher percentage of DRA assignment.

Figures 2–5 illustrate how covariates were associated with treatment assignment under the IV-OWL and OWL strategies, respectively. These figures are presented solely to demonstrate how an optimal treatment strategy can be visualized.

Figure 2 presents four heat maps for female participants, categorized by motor complication status: no motor complications, dyskinesias only, motor fluctuations only, and both dyskinesias and motor fluctuations. The y-axis and x-axis of each heat map represent the duration of levodopa use (years) and levodopa dosage (mg/day), respectively. Figure 3 is similar to Figure 2 but for male participants.

The heat maps revealed that the IV-OWL strategy was more likely to assign a DRA to female participants and an MAO-B inhibitor to male participants. Participants with dyskinesias were more likely to be assigned an MAO-B inhibitor. Furthermore, higher levodopa daily dosages were associated with an increased probability of being assigned a DRA, while longer durations of levodopa use were associated with a higher probability of being assigned an MAO-B inhibitor.

Figures 4, 5 are similar to Figures 2, 3 but illustrate how covariates were associated with treatment assignment under the OWL strategy. Like the IV-OWL strategy, the OWL strategy was more likely to assign an MAO-B inhibitor to participants with dyskinesias. However, unlike the IV-OWL strategy, male participants were more likely to be assigned a DRA, while female participants were more likely to receive an MAO-B inhibitor. In addition, the duration of levodopa use and levodopa daily dosage were not associated with treatment assignment under the OWL strategy.

Additional heat maps in Section B of the Appendix illustrate how treatment assignments vary across groups with different adverse events, such as nausea and insomnia. Readers can also explore our web tool to observe how treatment assignments change with variations in other covariates. The details regarding the use of the web tool can be found in Appendix C.

## Discussion

The IV-OWL strategy outperformed the OWL strategy and both benchmark strategies: the MAO-B inhibitor-only strategy and the DRA-only strategy. This finding is reasonable in the context of the NET-PD LS1 and PRECEPT/PostCEPT studies. In these trials, second-line therapy was often initiated between clinical visits, which limited the availability of covariate information measured close to the time of treatment initiation. As a result, unmeasured confounding is plausible. The IV-OWL strategy attempts to address potential unmeasured confounding using an instrumental variable, whereas the OWL strategy relies on the assumption of no unmeasured confounding. However, in settings where this assumption is more plausible, such as randomized treatment settings or studies with a rich set of covariates measured near treatment initiation, the OWL strategy may be preferred for its simplicity and efficiency.

To construct the IV-OWL strategy, it is important to identify a valid instrumental variable that satisfies Assumptions A1 – A3. In our study, we use the center’s treatment preference as an instrumental variable. However, not every center-level factor, nor center ID itself, should be considered a valid instrumental variable. For example, assessment protocols, rater variability, and differences in patient management may directly affect the outcome and, therefore, may violate the exclusion restriction. Thus, they would not be appropriate instrumental variables.

Another important consideration in constructing an optimal individualized treatment rule is selecting covariates for the decision rule. In this study, decision rules were developed to optimize motor function, as measured by the UPDRS III. Therefore, the covariates included in the rules were selected based on their relevance to motor outcomes and their availability in the harmonized data. More generally, the choice of covariates in an individualized treatment rule should be guided by the outcome or clinical objective being optimized. For example, if the goal is to minimize cognitive impairment or neuropsychiatric complications, then cognitive and neuropsychiatric measures should be included as candidate covariates. In practice, treatment decisions may require balancing multiple outcomes, including motor function, cognition, neuropsychiatric safety, and tolerability. In such settings, a composite outcome or multi-objective framework may be used, with covariates selected to reflect all relevant components of the clinical objective.

There are a few limitations in our study. Although the IV-OWL method outperformed other strategies, the improvement was not statistically significant. This result might be due to the small sample size and the lack of a rich set of covariates. Restricting the analysis to participants who initiated second-line therapy within 3 months of “baseline” resulted in a sample size of 84, while extending this period to 6 months increased it to only 121. The “baseline” covariates used to construct the treatment strategies were limited, and our choice of covariates was restricted to what was included in the NET-PD LS-1 and PRECEPT/PostCEPT trials. Previous studies of individualized therapy for PD have incorporated a broader range of patient characteristics, such as genetic and pharmacogenetic factors, as well as diverse medication types and administration methods (Titova and Chaudhuri, 2017; Mishima et al., 2021). Including additional covariates can improve the effectiveness of treatment decision rules. Additionally, our analysis of optimal choice of second-line therapy only included DRAs and MAO-B inhibitors and did not include catechol-O-methyltransferase inhibitors (rarely used in these trials) or adenosine A2A receptor antagonists (not available at the time of these trials). Finally, although we internally assessed the model using Monte Carlo cross-validation, external validation was not performed.

Future research with a richer set of covariates, a larger sample size, external validation datasets, and a broader range of second-line therapies is needed to develop more comprehensive and practical decision rules for second-line treatments for PD. To support such efforts, we have developed an R package for constructing optimal treatment strategies. The instructions to install the package can be found in Appendix C. Future work could also extend this framework to develop individualized second-line treatment regimens that simultaneously optimize motor outcomes and minimize adverse events. Finally, the proposed framework uses a linear kernel, which trades some flexibility for interpretability. An important direction for future research is to explore nonlinear extensions that improve model flexibility while preserving the explainability of the learned treatment rule.

## Conclusion

In this proof-of-concept study, we introduced two approaches for estimating individualized treatment strategies for patients with Parkinson’s disease: one that accounts for potential unmeasured confounding using an instrumental-variable framework, and one that assumes no unmeasured confounding. Both approaches were implemented using outcome-weighted learning with a weighted support vector machine (SVM). To demonstrate the practical use of these methods, we applied them to harmonized data from the NET-PD LS1 and PRECEPT/PostCEPT clinical trials. We estimated optimal individualized strategies for assigning second-line therapy, either a dopamine receptor agonist (DRA) or an MAO-B inhibitor, among participants already receiving levodopa.

A key strength of these methods is their interpretability. Because the estimated decision rules are linear, they can be visualized and inspected directly, allowing clinicians and researchers to better understand how patient characteristics influence treatment recommendations. This explainability feature may support the use of machine learning methods in clinical decision-making, where interpretability is often important.

In this paper, we illustrate how weighted SVM-based individualized treatment rules can be used to study treatment selection in Parkinson’s disease. These approaches may also be useful in other clinical settings where multiple treatment options are available, but guidance on individualized treatment selection remains limited. With further validation in larger studies, such methods may help support more personalized second-line therapy decisions and potentially improve outcomes for patients with Parkinson’s disease.

## Data Availability

The LS1 and Precept/POSTCEPT datasets have been made publicly available at the NINDS Archived Clinical Research Datasets.
