# Supplementary material for: An application of weighted support vector machines to individualized second-line therapy selection after levodopa in Parkinson’s disease
Source: Front Hum Neurosci. 2026 Jul 10;20:1859515. doi: 10.3389/fnhum.2026.1859515 (PMC13396011; doi:10.3389/fnhum.2026.1859515)
Supplement: Supplementary file 1 [file Supplementary_file_1.docx]

**Appendix for “An Application of Weighted Support Vector Machines to Individualized Second-Line Therapy Selection after Levodopa in Parkinson’s Disease” by Cuong T. Pham, Ruth B. Schneider, Charles S. Venuto, Greta Smith, Zachary P. Brehm, Michael P. McDermott, and Ashkan Erterfaie.**

**A. Doubly robust estimator of the value function**

The doubly robust estimator of the value function is given by

$$\begin{matrix} \mathcal{V}(\mathcal{D}) & =\frac{1}{n}\sum_{i=1}^{n} \frac{Z_{i}A_{i}Y_{i}I\left\{ A_{i}=\mathcal{D}\left( X_{i} \right) \right\}}{f\left( Z_{i}\mid X_{i} \right)\delta\left( X_{i} \right)}-\frac{Z_{i}\gamma^{'}\left( X_{i} \right)}{f\left( Z_{i}\mid X_{i} \right)\delta\left( X_{i} \right)} \\ & +\gamma\left( X_{i} \right)-\frac{Z_{i}\left( A_{i}-E\left[ A_{i}\mid Z_{i}=1,X_{i} \right] \right)}{2f\left( Z_{i}\mid X_{i} \right)\delta\left( X_{i} \right)}\gamma\left( X_{i} \right) \end{matrix}$$

where $\gamma^{'}(X)=E[AYI\{A=\mathcal{D}(X)\}\mid Z=-1,X]$ and $\gamma(X)=\sum_{z\in\{-1,1\}} zE[AYI\{A=\mathcal{D}(X)\}]/\delta(X)$.

We follow the recommendation from Cui et al. (13) to estimate the nuisance parameters. The nuisance functions $\delta(X)$ and $f(Z\mid X)$ are estimated using logistic regression models. The function $\gamma^{'}(X)$ is estimated using linear regression. To estimate $\delta(X)$, we fit a model that includes treatment assignment (DRA vs MAO-B inhibitor) as the dependent variable and the instrumental variable $Z$ as well as the covariates $X$ as the independent variables. Similarly, $f(Z\mid X)$ is fitted with the instrumental variable $Z$ as the dependent variable and the covariates $X$ as independent variables.

To estimate $\gamma^{'}(X)$, we fit a linear regression model with $AYI\{A=\mathcal{D}(X)\}$ as the dependent variable and $Z$ and $X$ being the independent variables to obtain the model for $E[AYI\{A=\mathcal{D}(X)\}\mid Z= -1,X]$.

To get the estimate for $\gamma(X)$, we solve the following doubly robust estimating equation:

$$\frac{1}{n}\sum_{i=1}^{n} \left[ A_{i}Y_{i}I\left\{ A_{i}=\mathcal{D}\left( X_{i} \right) \right\}-\gamma^{'}\left( X_{i} \right)-\frac{A-E\left[ A_{i}\mid Z_{i}=-1,X_{i} \right]\gamma\left( X_{i} \right)}{2} \right]\frac{Z_{i}}{f\left( Z_{i}\mid X_{i} \right)}=0$$

**B. Additional heat maps**


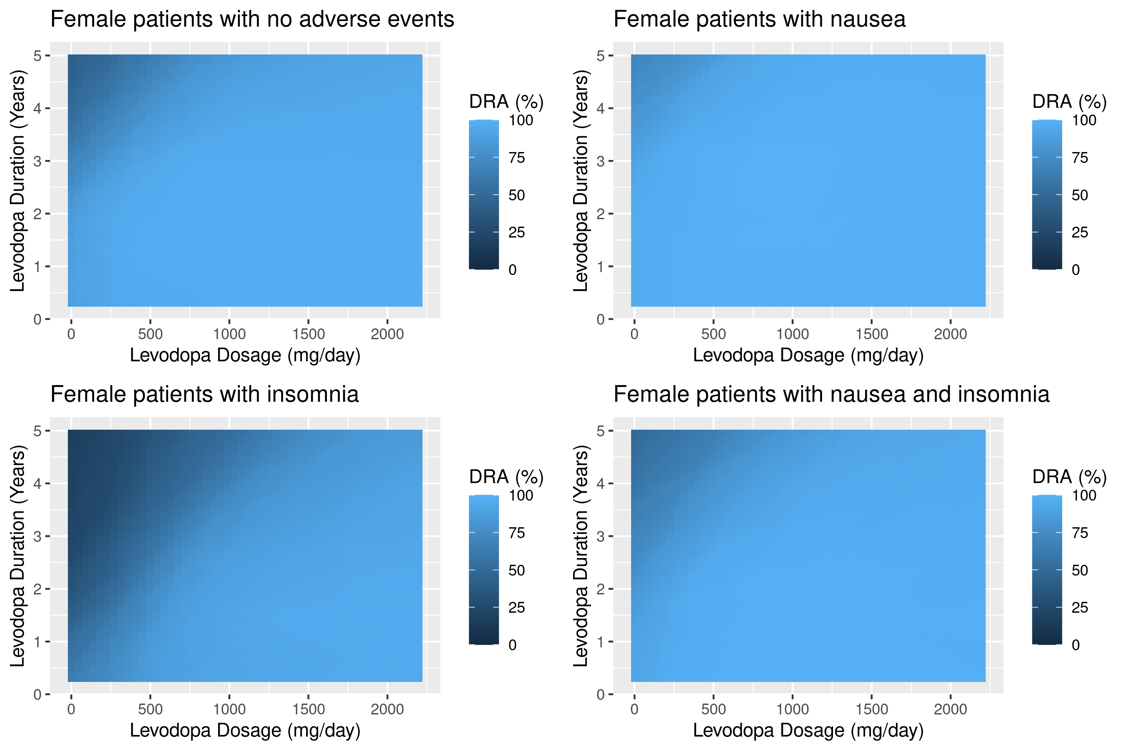


Figure 6: Heat maps showing the percentage of DRA treatment assignment for female subgroups based on levodopa dosage (mg/day), levodopa duration (years), and adverse events. Other covariates were held fixed at their reference or mean values. The decision rule is estimated using the IV-OWL method, with lighter blue indicating a higher percentage of DRA assignment.


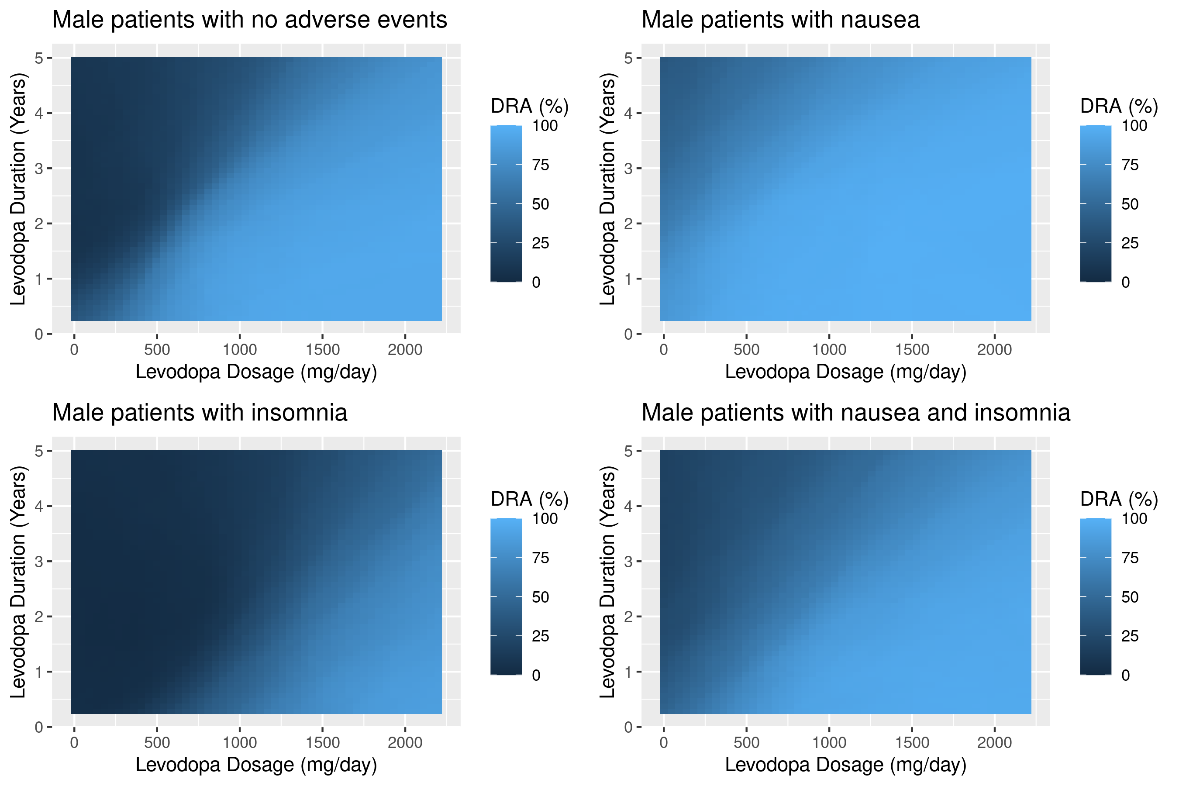


Figure 7: Heat maps showing the percentage of DRA treatment assignment for male subgroups based on levodopa dosage (mg/day), levodopa duration (years), and adverse events. Other covariates were held fixed at their reference or mean values. The decision rule is estimated using the IV-OWL method, with lighter blue indicating a higher percentage of DRA assignment.


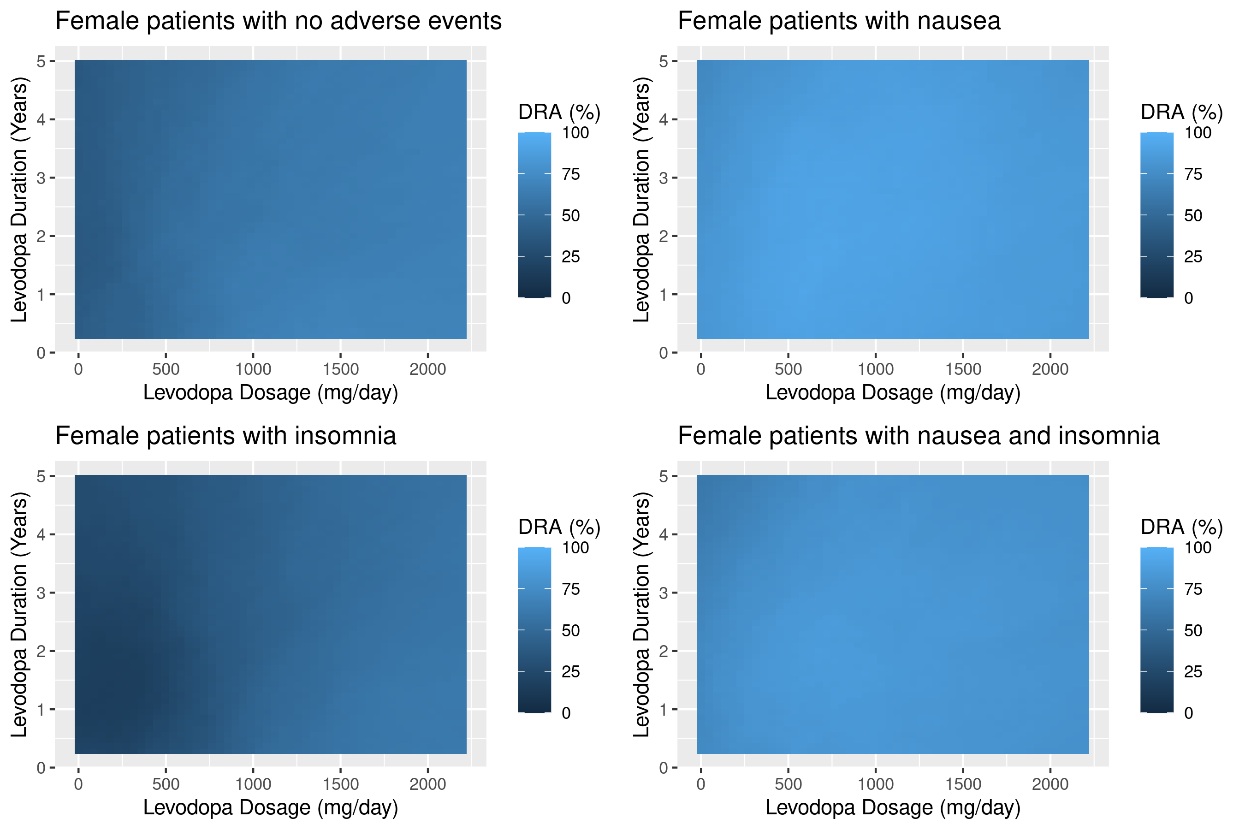


Figure 8: Heat maps showing the percentage of DRA treatment assignment for female subgroups based on levodopa dosage (mg/day), levodopa duration (years), and adverse events. Other covariates were held fixed at their reference or mean values. The decision rule is estimated using the OWL method, with lighter blue indicating a higher percentage of DRA assignment.


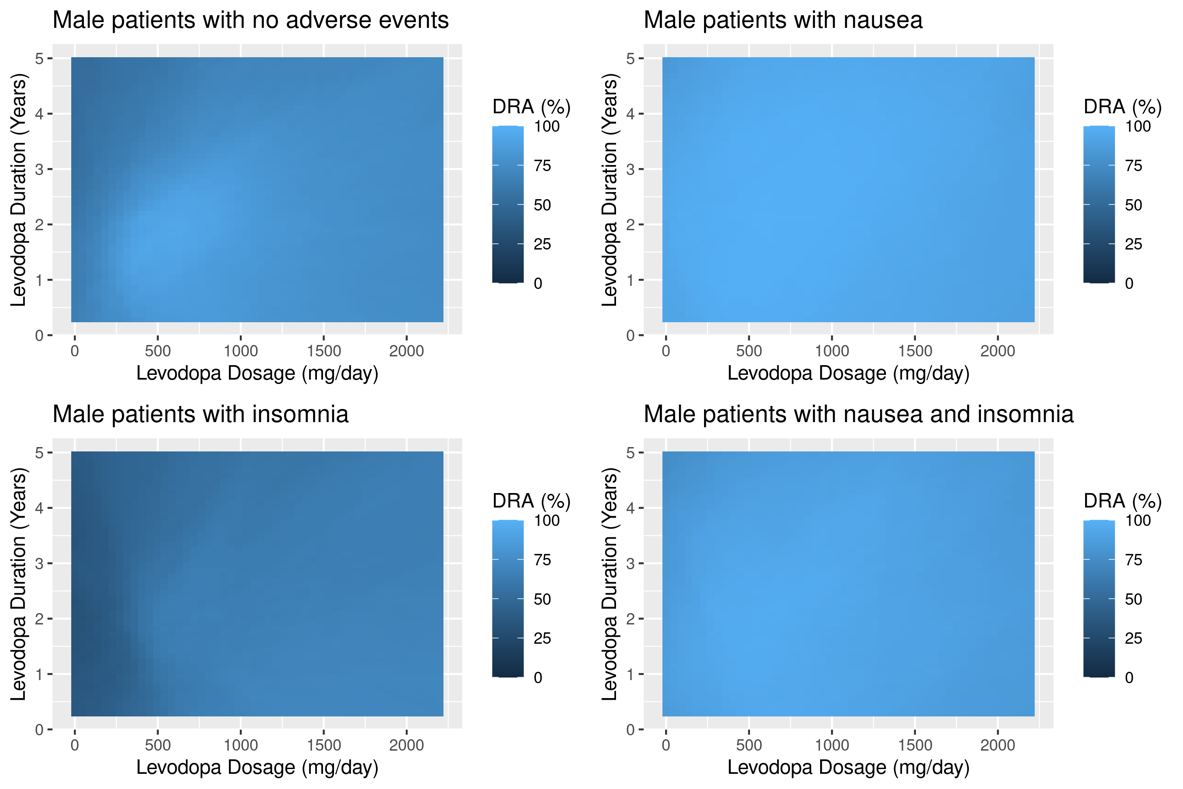


Figure 9: Heat maps showing the percentage of DRA treatment assignment for male subgroups based on levodopa dosage (mg/day), levodopa duration (years), and adverse events. Other covariates were held fixed at their reference or mean values. The decision rule is estimated using the OWL method, with lighter blue indicating a higher percentage of DRA assignment.

**C. R shiny app and R package**

**C.1 R shiny app**

We provide a Shiny app at the following link:

<https://q2mouj-cuong-pham.shinyapps.io/InteractiveVisualizationTool/>

The app demonstrates how the probability of treatment assignment of DRA varies with the “baseline” covariates: Levodopa dosage (mg/day), UPDRS I-III scores, time since diagnosis (years), duration of levodopa use (years), age (years), motor complications (dyskinesias, fluctuations), and adverse events (nausea, insomnia). Users can input specific covariates to study their impact on the treatment rule with the option to account for the assumption of unmeasured confounders. The tool generates a heat map displaying the percentages of DRA assignment across different values of the selected covariates.

**C.1 R package (ITR)**

The users can install the package from Github using the following command

devtools::install_github("cuongpham1995/ITR")

The package includes the two main functions:

- **fit.owl():** Fits an OWL model using a weighted SVM (wSVM) to optimize individual treatment rules. It calculates both IPW and MR estimators of value functions assuming that no unmeasured confounders exist.
- **fit.iv.owl():** Fits an IV Outcome-Weighted Learning model for individualized treatment strategy learning. It calculates both IPW and MR estimators of value functions assuming that unmeasured confounders exist.

For more detailed information on the inputs and outputs of the fit.owl() and fit.iv.owl() functions, the users can refer to the built-in documentation by typing **help(fit.owl)** and **help(fit.iv.owl)** in R after installing the package.
